# Large conductance voltage- and calcium-gated potassium channels (BK) in cerebral artery myocytes of perinatal fetal primates share several major characteristics with the adult phenotype

**DOI:** 10.1371/journal.pone.0203199

**Published:** 2018-09-13

**Authors:** Shivantika Bisen, Maria N. Simakova, Alex M. Dopico, Anna N. Bukiya

**Affiliations:** Department of Pharmacology, University of Tennessee Health Science Center, Memphis, Tennessee, United States of America; Cooper Medical School of Rowan University, UNITED STATES

## Abstract

Large conductance voltage- and calcium-gated channels (BK) control fundamental processes, including smooth muscle contractility and artery diameter. We used a baboon (*Papio spp*) model of pregnancy that is similar to that of humans to characterize BK channels in the middle cerebral artery and its branches in near-term (165 dGa) primate fetuses and corresponding pregnant mothers. In cell-attached patches (K^+^_pipette_ = 135 mM) on freshly isolated fetal cerebral artery myocytes, BK currents were identified by large conductance, and voltage- and paxilline-sensitive effects. Their calcium sensitivity was confirmed by a lower Vhalf (transmembrane voltage needed to reach half-maximal current) in inside-out patches at 30 versus 3 μM [Ca^2+^]_free_. Immunostaining against the BK channel-forming alpha subunit revealed qualitatively similar levels of BK alpha protein-corresponding fluorescence in fetal and maternal myocytes. Fetal and maternal BK currents recorded at 3 μM [Ca^2+^]_free_ from excised membrane patches had similar unitary current amplitude, and Vhalf. However, subtle differences between fetal and maternal BK channel phenotypes were detected in macroscopic current activation kinetics. To assess BK function at the organ level, fetal and maternal artery branches were pressurized *in vitro* at 30 mmHg and probed with the selective BK channel blocker paxilline (1 μM). The degree of paxilline-induced constriction was similar in fetal and maternal arteries, yet the constriction of maternal arteries was achieved sooner. In conclusion, we present a first identification and characterization of fetal cerebral artery BK channels in myocytes from primates. Although differences in BK channels between fetal and maternal arteries exist, the similarities reported herein advance the idea that vascular myocyte BK channels are functional near-term, and thus may serve as pharmacological targets during the perinatal-neonatal period.

## Introduction

Large conductance calcium- and voltage-gated potassium channels (BK, slo1 channels) control a plethora of physiological processes, including neuronal excitability, endocrine secretion, sensory function, and smooth muscle contractility [1]. Upon depolarization of the vascular myocyte membrane, smooth muscle BK channels generate outward potassium currents that bring the membrane potential back to resting levels and oppose vasoconstriction [2–4]. In vascular smooth muscle, BK channels have been reported to consist of a pore-forming alpha subunit and two types of accessory subunits: beta1 protein (BK beta1 subunit) and leucine-rich repeat containing protein 26 (BK gamma subunit) [1, 5–6]. Accessory proteins cannot form functional channels, but enable BK channel activation at lower transmembrane voltages when compared to homomeric channels formed by BK alpha subunit tetramers [7–8]. BK beta 1 subunits also modify the channel’s pharmacological profile by conferring, enhancing, or diminishing sensitivity to several endogenous and synthetic chemical regulators [9–13].

BK channels remain a constant focus of drug discovery, including BK targeting by newly developed compounds that modulate cerebral artery diameter and could potentially mitigate the vascular component of prevalent disorders in the perinatal period or adulthood, such as seizures, cerebrovascular lesions, stroke, migraines, and cerebral vasospasm [12, 14–16].

BK channel subunit composition and functional characteristics in cerebral arteries have been reported to vary from the fetal period to adolescence and early adulthood [17–19]. These studies, however, were performed in rodent and ovine subjects. Whether BK channels are present in the fetal primate vascular smooth muscle, and whether their functional characteristics differ from those present in the adult vasculature, remain unknown. In the current work, we addressed this gap in knowledge by studying BK channel subunit composition and the channel’s functional characteristics in freshly isolated myocytes from baboon cerebral arteries harvested at near-term pregnancy (165±3 days of gestation). Using patch-clamp recordings, immunofluorescence staining of BK channel protein, and pressurized artery diameter monitoring *in vitro*, we were able to identify both BK channel protein expression and an ion current phenotype in fetal baboon cerebral artery smooth muscle that fulfill key criteria for BK channel activity. Moreover, these functional BK channels contribute to the regulation of cerebral artery diameter. Comparison of near-term fetal BK currents with that of adult mother baboons revealed several similar functional features.

## Materials and methods

### Ethical approval

Care of animals and experimental protocols were reviewed and approved by the Institutional Animal Care and Use Committee of the University of Tennessee Health Science Center, which is an institution accredited by the Association for Assessment and Accreditation of Laboratory Animal Care International (AAALACi).

### Animal subjects

A total of seven *Papio hamadryas anubis* dams (ages 7–15 yrs) were used. Dams were singly housed in standard baboon cages, with visual and audio access to each other. Baboons were on a 12-hour light/dark cycle (lights on at 6:00 am) without access to natural light. Feeding was performed twice a day, each consisting of High Protein Monkey Diet (~15 biscuits per meal, 21 kcal/biscuit) to sustain baboon’s weight gain as expected throughout the pregnancy. Each feeding was also supplemented by two pieces of fresh fruits or vegetables and two table spoons of peanut butter. Drinking water was available *ad libitum*. Facilities were maintained in accordance with the USDA and AAALAC standards.

Cesarean sections were performed at 165±3 days of gestation, as confirmed by a Doppler sonography evaluation. This gestational age is near-term in baboons [20], and results in viable neonates [21]. Cesarean section surgery was performed using standard methodology that included maternal anesthesia (induction with ketamine hydrochloride, 10 mg/kg of body weight, and maintenance with 1.5–2% isoflurane). Six female fetuses and one male fetus were delivered. During c-section, fetuses were euthanized by exsanguination while still under the influence of maternal anesthesia. Mothers were euthanized by a single injection of Euthasol^®^ (sodium pentobarbital) through the uterine or ovarian vein following the removal of the placenta and contraction of the uterus. In all biochemical experiments, maternal and fetal tissue samples were processed simultaneously.

### Myocyte isolation from cerebral artery tissue

Immediately following fetal and maternal euthanasia, middle cerebral arteries and their 1^st^ and 2^nd^ order branches were dissected out and placed into their respective plates filled with ice-cold dissociation medium (DM) with the following composition (mM): 0.16 CaCl_2_, 0.49 EDTA, 10 HEPES, 5 KCl, 0.5 KH2PO_4_, 2 MgCl_2_, 110 NaCl, 0.5 NaH_2_PO_4_, 10 NaHCO_3_, 0.02 phenol red, 10 taurine, and 10 glucose; pH = 7.4. Each artery segment was cut into 1- to 2-mm long rings (up to 30 rings/experiment). Individual myocytes from maternal and fetal artery segments were enzymatically isolated at the same time using a two-step protocol. During the first enzymatic step, artery segments were placed into 3 ml DM containing 0.03% papain, 0.05% bovine serum albumin (BSA), and 0.004% dithiothreitol at 37°C for 15 minutes in a polypropylene tube and incubated in a shaking water bath (37°C, 60 oscillations/min). For the second step, the supernatant was discarded, and the tissue was transferred to a polypropylene tube with 3 ml of DM containing 0.06% soybean trypsin inhibitor (STI), 0.05% BSA, and 2% collagenase (26.6 units/ml) (Sigma-Aldrich, St. Louis, MO). The tube was incubated again in a shaking water bath at 37°C and 60 oscillations/min for 15 minutes. Finally, the artery tissue pellet was transferred into a tube with 3 ml of DM containing 0.06% STI for mechanical isolation of myocytes. In particular, tissue-containing DM was pipetted using a series of borosilicate Pasteur pipettes having fire-polished, diminishing internal diameter tips. The procedure rendered a cell suspension containing relaxed, individual myocytes (2–3 myocytes/field using a 20× objective) that could be identified under an Olympus IX-70 microscope (Olympus American Inc., Woodbury, NY). The cell suspension was stored in ice-cold DM containing 0.06% STI and 0.06% BSA. Myocytes were used for electrophysiology and immunofluorescence staining up to 4 hours after isolation.

### Electrophysiology data acquisition

BK currents were recorded from cell-attached or excised, inside-out (I/O) membrane patches; the configuration is stated in the Results. On each experimental day, recordings from maternal and fetal myocytes were performed in alternate order. Both bath and electrode solutions contained (mM): 130 KCl, 5 EGTA, 2.44 MgCl_2_, 15 HEPES, and 1.6 HEDTA; pH = 7.35. The calcium level was adjusted by the addition of 1 mM CaCl_2_ stock solution to render [Ca^2+^]_free_≈3 or 30 μM. Nominal free Ca^2+^ was calculated with MaxChelator Sliders (C. Patton, Stanford University, CA) and validated experimentally using Ca^2+^- selective electrodes (Corning Incorporated Science Products Division, Corning, NY). Patch-clamp electrodes were pulled from glass capillaries (Drummond Scientific Co.). Immediately before recording, the tip of each electrode was fire-polished on a microforge WPI MF-200 (World Precision Instruments, Sarasota, FL) to give resistances of 5–9 MΩ when filled with electrode solution. An agar bridge with Cl^-^ as the main anion was used as a ground electrode. Experiments were performed at room temperature (20°–22°C). The ionic current was recorded using an EPC8 amplifier (HEKA, Lambrecht, Germany) at 1 kHz. Data were digitized at 5 kHz using a Digidata 1320A A/D converter and pCLAMP 8.0 (Molecular Devices, Sunnyvale, CA). In each patch, high transmembrane voltages (≥80 mV) were used to render maximal/near-maximal channel activity.

For macroscopic current recordings, currents were evoked by a series of 200 ms-long voltage steps of 10 mV from -150 to +170 mV (Vholding = -80 mV). During macroscopic current recordings, a P/4 leak subtraction routine was applied using a built-in function in pCLAMP.

### Immunocytochemistry and confocal fluorescence imaging

Staining procedures were performed on two occasions: each included parallel staining of myocytes from mothers and their corresponding fetuses. Isolated myocytes were dispersed on a coverslip, left to settle for 45–60 min, and then fixed in 3% paraformaldehyde at room temperature for 30 min. Upon paraformaldehyde washout, the specimen was permeabilized with 0.1% Triton-100 in phosphate buffered saline at room temperature for 30 min.

Specimens were incubated at 4°C overnight in mouse monoclonal antibody against BK alpha subunit (clone L6/60, UC Davis/NIH NeuroMab Facility, Davis, CA) at 1:200 dilution. After primary antibody washout, arteries were incubated in anti-mouse secondary antibody conjugated with Alexa488 (A11001, Invitrogen, Carlsbad, CA) at 1:1,000 dilution, at room temperature for 2 hrs. Staining with secondary antibody in the absence of primary antibody was used as a negative control. Slips were mounted using ProLong AntiFade kit (Invitrogen, Carlsbad, CA) and sealed using clear nail polish.

The acquisition settings of the confocal microscope system remained unchanged throughout imaging of all specimens. Myocytes were imaged using 60x objective and 488 (Alexa488) laser line using a z-stack option (1 μm step) of the Olympus FV-1000 laser scanning confocal system (Center Valley, PA) at the Department of Pharmacology Confocal Imaging Facility (UTHSC).

### Anti-BK alpha subunit antibody performance validation by Western blot

Baboon fetal and maternal cerebral artery segments were dissected out and subjected to surface protein biotinylation using the Pierce^™^ Cell Surface Protein Isolation Kit (Thermo Fisher Scientific, Waltham, MA), following manufacturer’s instructions. Surface (extracellular) versus intracellular protein was analyzed by Western blotting using standard methodology as described [22]. The presence of a single band in both protein fractions was detected following staining with mouse monoclonal anti-*KCNMA1* antibody (1:1,000 dilution; clone L6/60, UC Davis/NIH NeuroMab Facility). Additional validation was performed on rat and C57BL/6 mouse cerebral artery lysate (positive controls) versus lysate of non-transfected human embryonic kidney (HEK) cells and *KCNMA1* global knockout mouse cerebral artery (negative controls). The total protein amount loaded was the same in all samples within each blot as determined by using the BCA protein assay kit (Thermo Fisher Scientific, Waltham, MA). For positive and negative control blots, staining against beta-actin (mouse monoclonal anti-beta-actin antibody, 1:1,000 dilution; ab8226, Abcam) was used to validate successful loading of the sample. In the case of baboon cerebral artery blots, intracellular versus surface protein fractions were separated using the Cell Surface Protein Isolation kit following manufacturer’s instructions (Thermo Fisher Scientific, Waltham, MA). Samples representing separate protein fractions were loaded into different gels, two gels per fraction. While one gel was subjected to transfer and Western blotting, another one was stained with coomassie blue to serve as an independent verification of loaded protein amount.

### qPCR

qPCR for LRRC26 (F gctgcgcaacctctcatt, R tgtcctgcaggctgagtg) was performed as described in our earlier publication [23].

### Cerebral artery diameter monitoring

7–12 mm-long artery segments were cannulated at each end in a temperature-controlled, custom-made perfusion chamber. Using a Dynamax RP-1 peristaltic pump (Rainin Instr.), the chamber was continuously perfused at a rate of 3.75 ml/min with physiologic saline solution (PSS) that contained (mM): 119 NaCl, 4.7 KCl, 1.2 KH_2_PO_4_, 1.6 CaCl_2_, 1.2 MgSO_4_, 0.023 EDTA, 11 glucose, and 24 NaHCO_3_. PSS was continuously equilibrated at pH 7.4 with a 21/5/74% mix of O_2_/CO_2_/N_2_ and maintained at 35–37°C. Arteries were monitored with a CCD camera (Sanyo VCB-3512T) attached to an inverted microscope (Nikon Eclipse TS100). The artery external wall diameter was measured using the automatic edge-detection function of IonWizard software (IonOptics) and digitized at 1 Hz. Steady-state changes in intravascular pressure were achieved by elevating an attached reservoir filled with PSS and were monitored using a pressure transducer (Living Systems Instr.). Arteries were first incubated at an intravascular pressure of 10 mm Hg for 10 min. Then, intravascular pressure was increased to 30 mm Hg and held steady throughout the experiment to evoke development and maintenance of arterial myogenic tone. For testing functionality of the artery contractile machinery, a high-KCl solution was used, which consisted of (mM): 63.7 NaCl, 60 KCl, 1.2 KH_2_PO_4_, 1.6 CaCl_2_, 1.2 MgSO_4_, 0.023 EDTA, 11 glucose, and 24 NaHCO_3_. As done with regular PSS, the high-KCl solution was continuously equilibrated at pH 7.4 with a 21/5/74% mix of O_2_/CO_2_/N_2_, and maintained at 35–37°C. At the end of each experiment, arteries were probed with calcium-free PSS as described in our previous publications [13, 24]. Artery dilation in response to calcium-free solution was used as a parameter of arterial segment viability.

### Chemicals

Chemicals were purchased from Sigma-Aldrich (St. Louis, MO). Paxilline was first diluted in dimethyl sulfoxide (DMSO) to render 22.9 mM stock. Stock was further diluted in PSS to render 1 μM paxilline.

### Data analysis

For patch-clamp data, the product of the number of channels in the patch (N) and channel open probability (Po) was used as an index of channel steady-state activity. NPo was obtained using a built-in option in Clampfit 9.2 (Molecular Devices) from ≥20 seconds of gap-free recording under each condition. For determination of Vhalf, NPo/NPomax-V and G/Gmax-V plots were fitted using a built-in Boltzmann fitting function in Origin 7.0 (Originlab Corp). The τ_act_ values were calculated based on exponential fitting of the records using a built-in fitting function in Clampfit 9.2.

Fluorescence signals were quantified along myocyte plasma membranes as visually defined upon the superposition of fluorescence with visible light images at the middle of the z-stack. This approach allowed capturing of a sharp fluorescence signal along the myocyte plasma membrane. The background fluorescence was measured outside of the myocytes and subtracted from fluorescence intensity of the specimen. Mean pixel intensity is reported.

Artery diameter data were analyzed using IonWizard 4.4 software (IonOptics). Myogenic tone was determined using the following formula: (1-artery diameter at 45 min following application of the test pressure/maximum diameter that was observed during application of the test pressure)X100. The effect of drug applications was evaluated at the time it had reached a maximal, steady level. If apparent fluctuations of diameter during drug application were observed, the average of the diameter during the second half of drug application was used as a data point, reflecting the drug’s effect.

Final plotting, fitting, and statistical analysis of the data were conducted using Origin 8.5 (OriginLab, Northampton, MA) and InStat 3.0 (GraphPad, La Jolla, CA). Normal distribution of datasets with the number of observations exceeding 10 was assessed by the Kolmogorov-Smirnov test: unless stated otherwise, such datasets were assumed to follow a Gaussian distribution. For smaller datasets (<10) and large datasets that did not pass the Kolmogorov-Smirnov test, statistical analysis was performed using the non-parametric Mann-Whitney test. On datasets that followed normal distributions, statistical analyses were conducted using unpaired Student’s t-test with two-tail P value. In all cases, P< 0.05 was considered statistically significant. Data are expressed as the mean ± S.E.M. The number of individual observations for each dataset is provided in the figure legends.

## Results

### Identification of BK current in freshly isolated cerebral artery myocytes of fetal baboons

**Fig 1:**
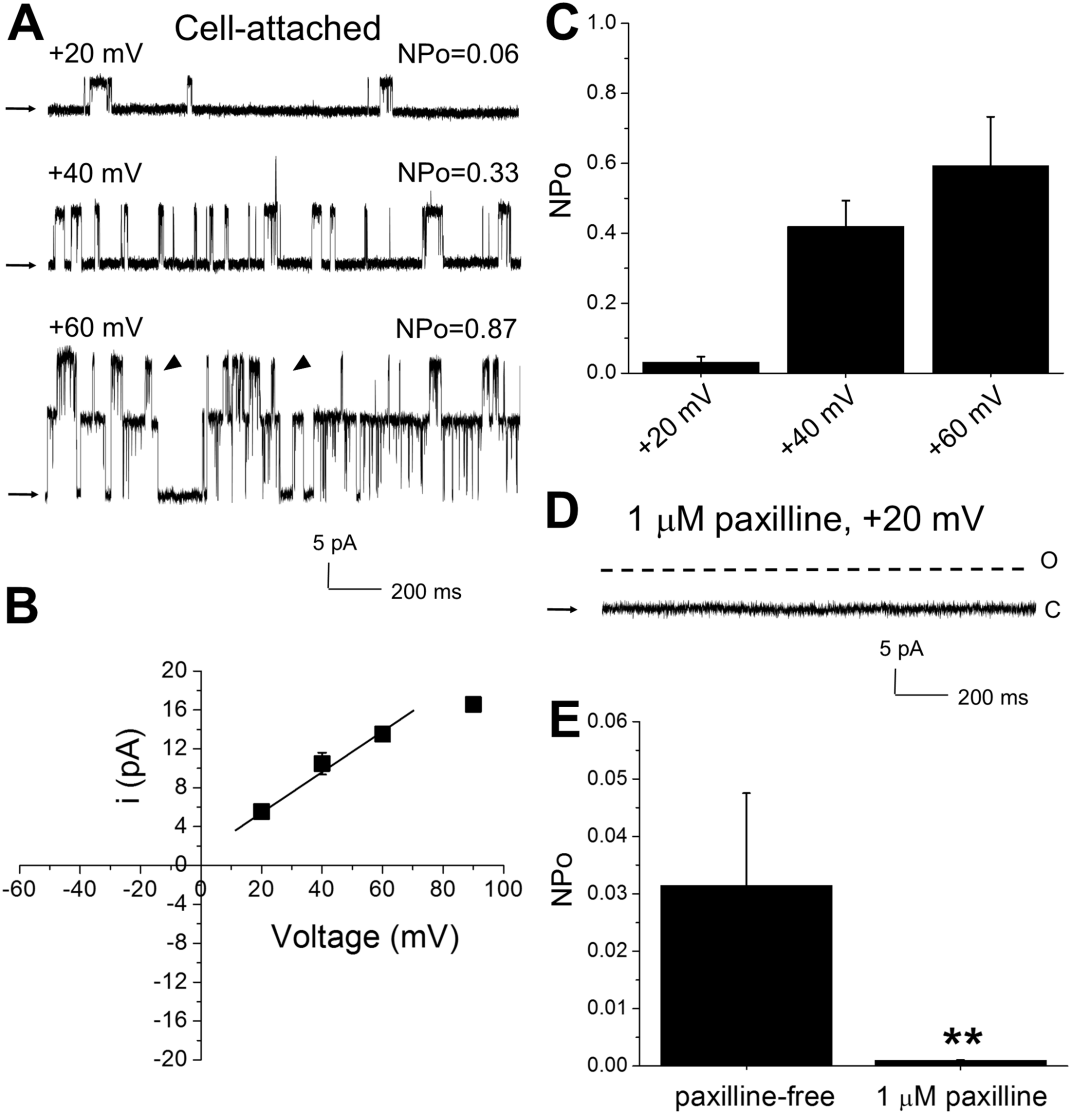
Large amplitude voltage- and paxilline-sensitive currents are detected in cell-attached patches in freshly isolated cerebral artery myocytes of fetal baboon. **A.** Original records showing large conductance current at different voltages. Arrows pointing to baseline indicate all channels closed. Channel openings of low unitary current amplitude are highlighted by pointed triangles. NPo = number of opening (N) x open probability. NPo represents open probability of all observed opening levels. **B.** Averaged unitary current amplitude as a function of holding voltage. Here and in **C**, values represent averaged data of 3 cell-attached patches that were obtained from different fetuses. Linear fit was obtained using built-in function in Origin 8.5.1 (OriginLab, Northampton, MA); adjusted R-Square = 0.96814. The fitting excludes a data point at high voltage, as unitary current amplitudes at high positive voltages are known to deviate from linear behavior [65–66]. **C.** Averaged NPo at different holding voltages. In this plot, NPo at +60 mV is only calculated for large conductance channel. **D.** Original trace showing lack of current in presence of selective BK channel blocker 1 μM paxilline in the patch pipette. O: opening level; C: closure level. **E.** Bar graph shows significant decrease in current in presence of paxilline. n = 3 at each experimental condition; patches within each experimental condition were obtained from different fetuses. **Statistically significant difference from paxilline-free pipette solution, P = 0.0056.

Cell-attached patch recordings (pipette [K^+^] = 135 mM) from freshly isolated cerebral artery myocytes of near-term fetuses showed two types of channel open amplitudes, with the smaller becoming apparent at higher levels of membrane depolarization (e.g. +60 mV) in 2 out of 3 patches (Fig 1A). This current reached on average 9.5±0.2 pA in near-symmetric K^+^_o_/K^+^_i_ gradient at 60 mV. Considering the large conductance of BK channels, we focused on the ion channel events with larger unitary amplitude (e.g. 14.0±0.3 pA in near-symmetric K^+^_o_/K^+^_i_ gradient at +60 mV). Thus, single current amplitude (i)-voltage relationship data from cell-attached patches were fitted to a linear function that yielded a mean slope conductance of 219±37 pS (Fig 1B). The channels under study exhibited a clear voltage dependence: NPo was drastically increased when the membrane was depolarized from a holding potential of 0 mV by voltage steps ranging from +20 to +60 mV (Fig 1C). The voltage-dependent, large conductance ion channel was also probed with the specific BK channel blocker paxilline [25]. Addition of 1 μM paxilline [26] to the patch pipette solution led to an inability to detect the current (Fig 1A **versus** Fig 1D and 1E), revealing a hallmark pharmacological feature of BK channels.

While the term “BK channel” has been usually applied to the calcium-gated (slo1) channel protein, a variety of voltage-dependent K^+^ permeable channels, such as slo2 or slo3, are also characterized by large unitary conductances. Most of them, however, are gated by ions other than calcium [27–28]. To determine whether the observed current in fetal baboon myocytes possessed the characteristic calcium-sensitivity of slo1 channels, we obtained excised inside-out (I/O) patches and exposed their intracellular side to 3 and 30 μM [Ca^2+^]_free_. These calcium levels are reached in the vicinity of native BK channels in cerebral arteries during myocyte contraction [29].

**Fig 2:**
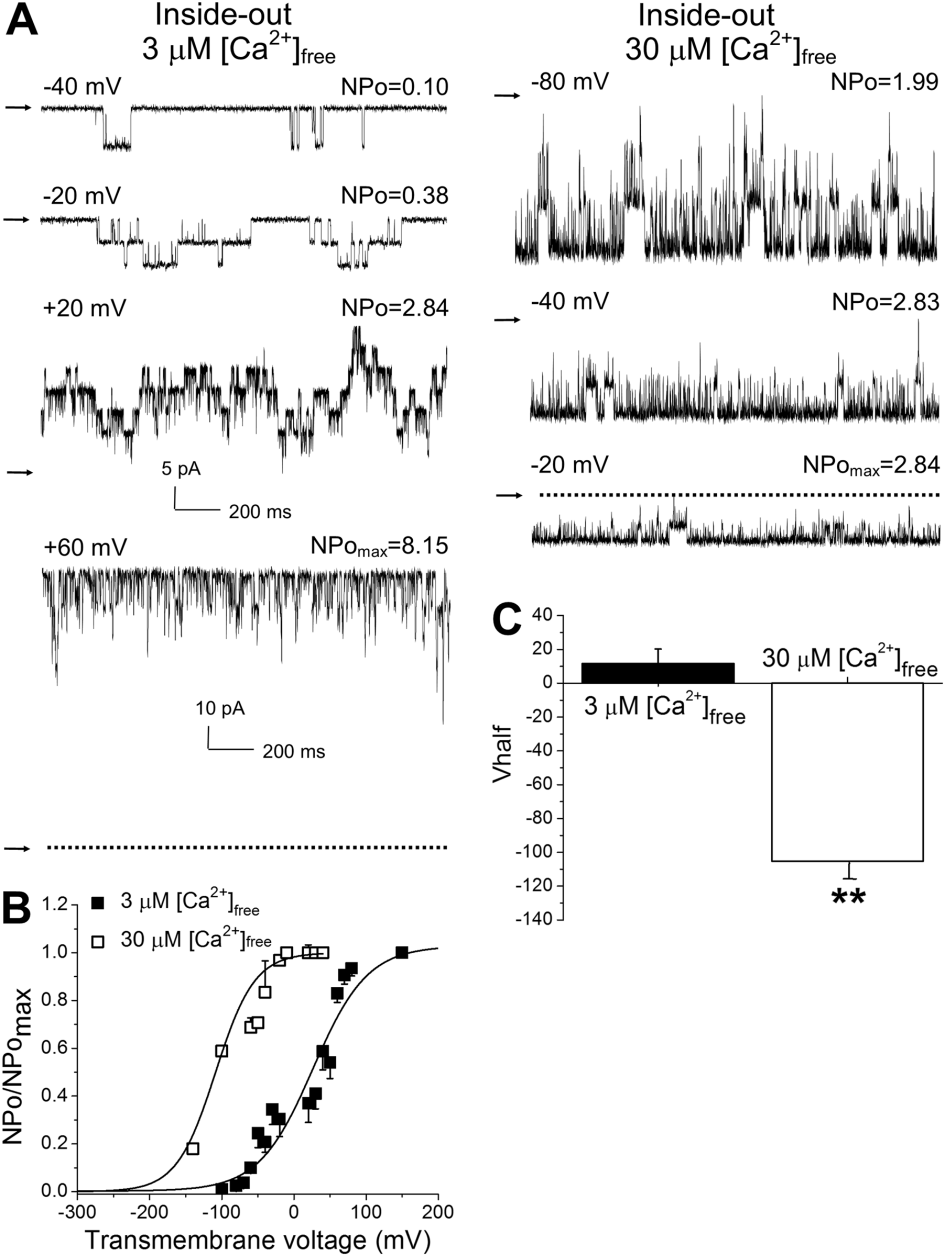
Calcium-sensitive behavior of the current is confirmed in excised (inside-out) patches from freshly isolated cerebral artery myocytes of fetal baboon. **A.** Original recordings of channel activity at different transmembrane voltages in I/O patches at 3 μM and 30 μM [Ca^2+^]_free_ bathing of the intracellular side of the patch membrane. Arrows pointing to baseline indicate all channels closed. NPomax = maximal open probability detected for this membrane patch. Please note that current amplitude scale for +60 mV recording obtained at 3 μM [Ca^2+^]_free_ differs from the scale for other records. **B.** Averaged NPo/NPomax-V curves show leftward shift of the current along the voltage axis at 30 μM when compared to 3 μM [Ca^2+^]_free_. Here and in **C**, each averaged data point was obtained from 3 patches at 30 μM (each patch obtained from a different fetus) and 14 patches at 3 μM [Ca^2+^]_free_ (each patch was obtained from a different myocyte, no more than 5 patches were obtained from the artery of one fetus). **C.** Averaged Vhalf (voltage that is needed to reach half-maximal current) at 30 μM [Ca^2+^]_free_ is significantly lower than Vhalf at 3 μM [Ca^2+^]_free_. **Statistically significant difference from 3 μM [Ca^2+^]_free_, P = 0.0001.

At 3 μM [Ca^2+^]_free_, averaged Vhalf was 11±9 mV (Fig 2). As [Ca^2+^]_free_ was increased to 30 μM, the NPo/NPomax-V curve shifted to less depolarized values, with averaged Vhalf decreasing to -105±10 mV (Fig 2). This decrease in Vhalf was so apparent that statistical significance was achieved after testing 3 patches. Overall, the observed physical and pharmacological characteristics of the current, such as large conductance, and voltage-, calcium-, and paxilline-sensitive behavior, indicated that the phenotype under investigation in fetal cerebral artery myocytes matched that of BK channels.

### Fetal cerebral artery myocyte BK channels exhibit protein and current features that are similar to those of BK channels from maternal cerebral artery myocytes

**Fig 3:**
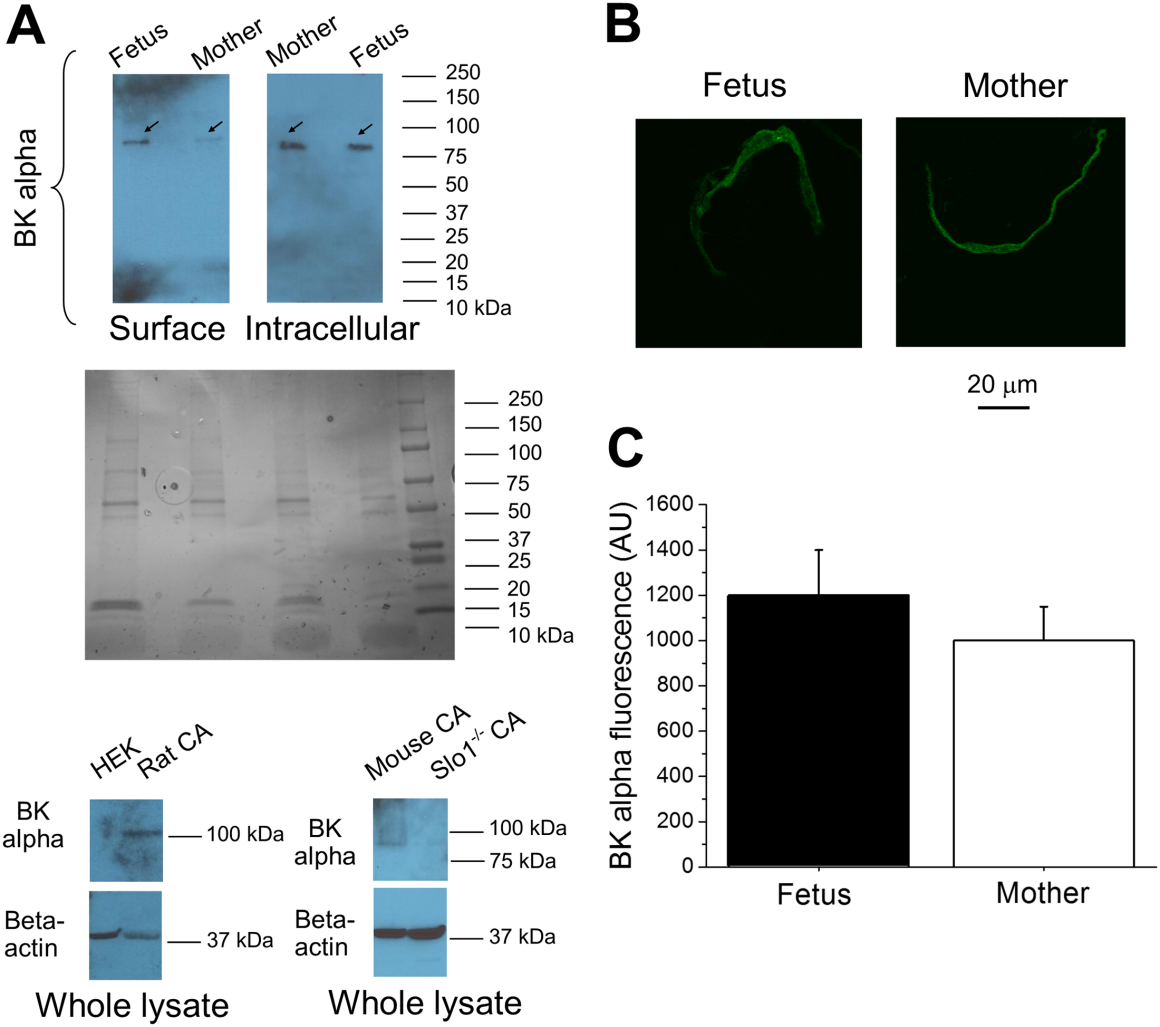
BK channel-forming alpha subunit in cerebral artery myocytes of fetal baboons and their mothers as detected by immunofluorescence labeling. **A.** Western blots validating antibody performance in surface biotinylated fetal and maternal artery segments. In each column, arrows point to a single band following Western blot procedure with anti-*KCNMA1* antibody. Black-and-white insert depicts gels stained with coomassie blue to verify protein load of baboon samples. Whole-cell lysates of non-transfected HEK cells (left) and cerebral arteries of *KCNMA1* (slo1^-/-^) (right) mouse served as negative controls; whole lysates of rat (left) and C57BL/6 mouse (right) cerebral arteries served as positive controls. CA: cerebral artery. **B.** Snapshots of fetal baboon and maternal cerebral artery myocytes following immunofluorescence staining with anti-BK channel alpha antibody. **C.** Averaged data showing BK alpha subunit-associated fluorescence signal in fetal and maternal myocytes (P = 0.1216). Myocyte membrane area was determined upon superposition of fluorescence images with myocyte images in visible light spectrum. Fetal data were obtained from 28 myocytes of 2 fetuses; maternal data were obtained from 19 myocytes of 2 mothers. AU: arbitrary unit. Here, and in Figs 4–6, fetal data are depicted by black symbols/bars, while maternal data are presented by hollow symbols/bars.

First, we designed the experiment to detect the presence of BK channel subunit protein in fetal and maternal cerebral artery myocytes. For this purpose, we started with identifying band(s) on Western blot that is(are) likely to be associated with BK channel alpha subunit protein. In fetal and maternal baboon cerebral arteries following surface protein biotinylation, a single band appeared between 80 and 100 kDa (Fig 3A). Bands of similar molecular weight appeared in Western blots of cerebral artery lysates from C57BL/6 mouse but not in artery lysates from *KCNMA1* (e.g. slo1-lacking) global knockout mouse on C57BL/6 background [30]. However, the use of the same antibody in our previous work on rat cerebral arteries rendered a band of ≈100–130 kDa [31]. In the current work, we repeated the experiment and obtained a ≈100–110 kDa band from rat cerebral artery lysates, this band being absent in lysate of non-transfected HEK293 cells (Fig 3A). Thus, slight variations in the observed molecular weight of the band are likely explained by differences between species, while the detected band in baboon cerebral arteries very likely corresponds to the BK alpha protein. We refrained from quantifying band intensity in Western blots, because bands were obtained from whole artery samples. Although over half of cerebral artery cellular content is presented by smooth muscle cells [32], the presence of BK channel alpha subunit protein in other cellular types (e.g. endothelium) cannot be excluded. Therefore, we proceeded with immune labeling of isolated myocytes, where cellular identity of the staining is certain. Maternal myocytes were isolated following the same procedure that was used for fetal cerebral artery myocyte isolation. Freshly isolated myocytes were immune-stained with anti-BK alpha subunit antibody that rendered the single band in Western blots (Fig 3A). Fluorescence intensity analysis following the immunostaining against BK alpha subunit protein did not render a statistically significant difference between fetal and maternal specimens (Fig 3B and 3C). Unlike BK alpha, we were unable to obtain accurate staining against BK beta1 and BK gamma subunits due to inconsistent performance of corresponding antibodies. Yet, based on observations from other groups, presence of BK beta1 subunits in primate cerebral artery myocytes is highly likely (see Discussion). With regards to LRRC, quantitative PCR analysis of samples from 4 fetuses and 4 mothers yielded signals corresponding to LRRC26 mRNA, in which concentrations did not differ significantly between fetal (1.6±0.2) versus maternal (1.6±0.1) preparations (P = 0.6883).

**Fig 4:**
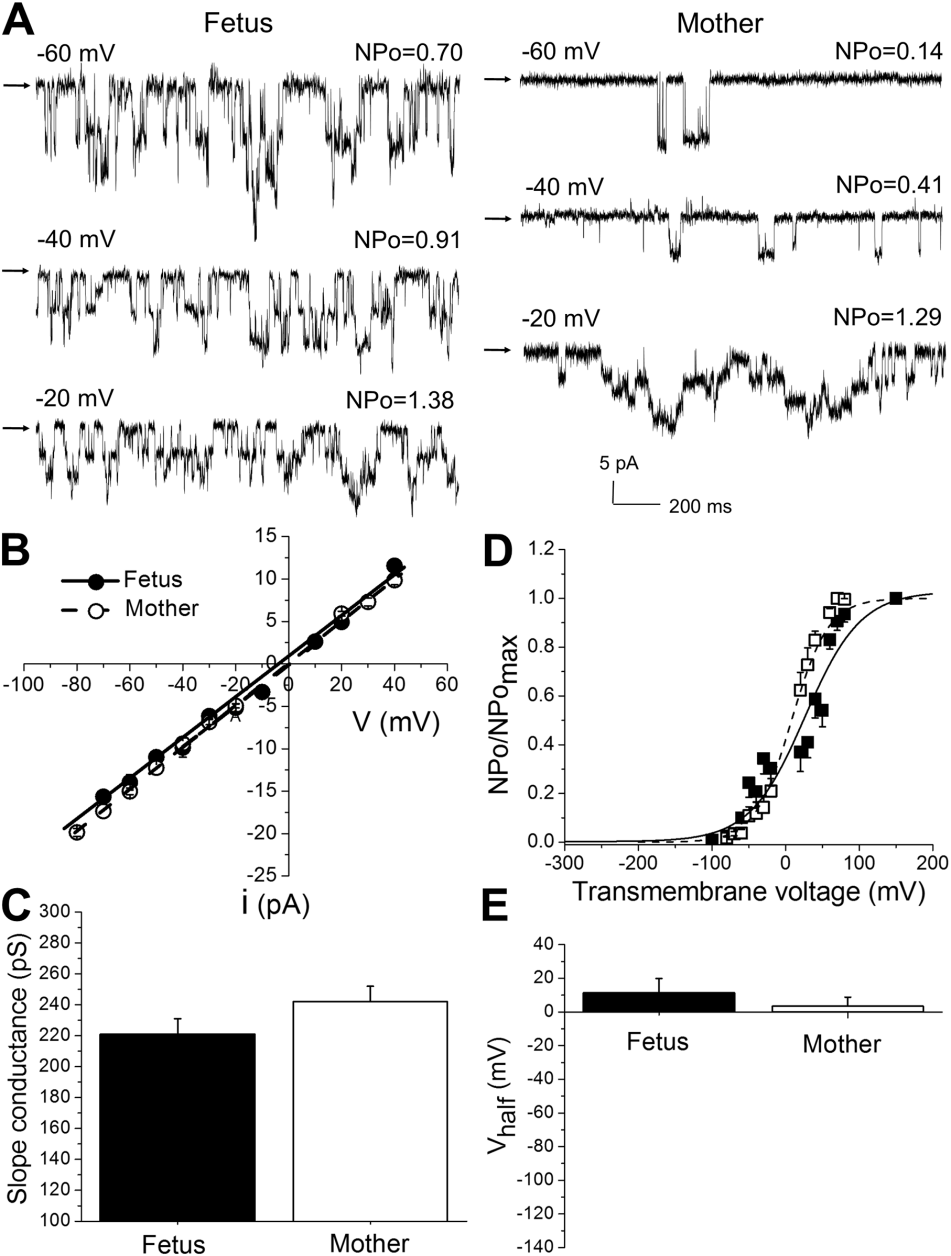
BK currents in excised I/O patches from cerebral artery myocytes of fetal baboons and their mothers. **A.** Original recordings of BK currents in I/O patches from fetal and maternal cerebral artery myocytes at 3 μM [Ca^2+^]_free_ at different transmembrane voltages. Arrows pointing to baseline indicate all channels closed. In patch from fetal myocyte, NPomax = 4.68, from maternal myocyte NPomax = 3.9 mV. **B.** Averaged unitary current as a function of transmembrane voltage. Linear fits were obtained after averaging data from 9 patches of myocytes from 4 fetuses and 7 patches of myocytes from 4 mothers. Each patch was obtained from a separate myocyte. **C.** Averaged slope conductances are similar in fetal and maternal BK channels (symmetric K^+^ = 135 mM; P = 0.1841), number of observations is same as in **B**. **D.** Averaged NPo/NPomax-V curves comparing fetal and maternal currents at 3 μM [Ca^2+^]_free_. **E.** Averaged Vhalf values at 3 μM [Ca^2+^]_free_ are similar in fetal (n = 14) and maternal (n = 14) BK currents (P = 0.4565). Fetal data were obtained from 14 patches of myocytes from 4 fetuses and 14 patches of myocytes from 4 mothers. Each patch was obtained from a separate myocyte.

Considering that BK alpha monomer levels estimated with an antibody-based approach cannot predict expression of a functional BK channel, we proceeded to conduct electrophysiological studies. First, we compared fetal and maternal BK current characteristics in I/O patches across a wide range of transmembrane voltages (from -80 to +40 mV) at a physiologically relevant level of calcium (3 μM [Ca^2+^]_free_) to evaluate functional characteristics of BK channels, and therefore infer their subunit composition. The identity of the maternal current was verified by its large conductance of 242±10 pS (symmetric K^+^ = 135 mM), which was similar to the averaged slope conductance of the fetal BK current of 221±10 pS under the same experimental conditions (Fig 4A–4C). This result indicates that channel permeation by K^+^ is similar between the maternal and the fetal BK channels.

**Fig 5:**
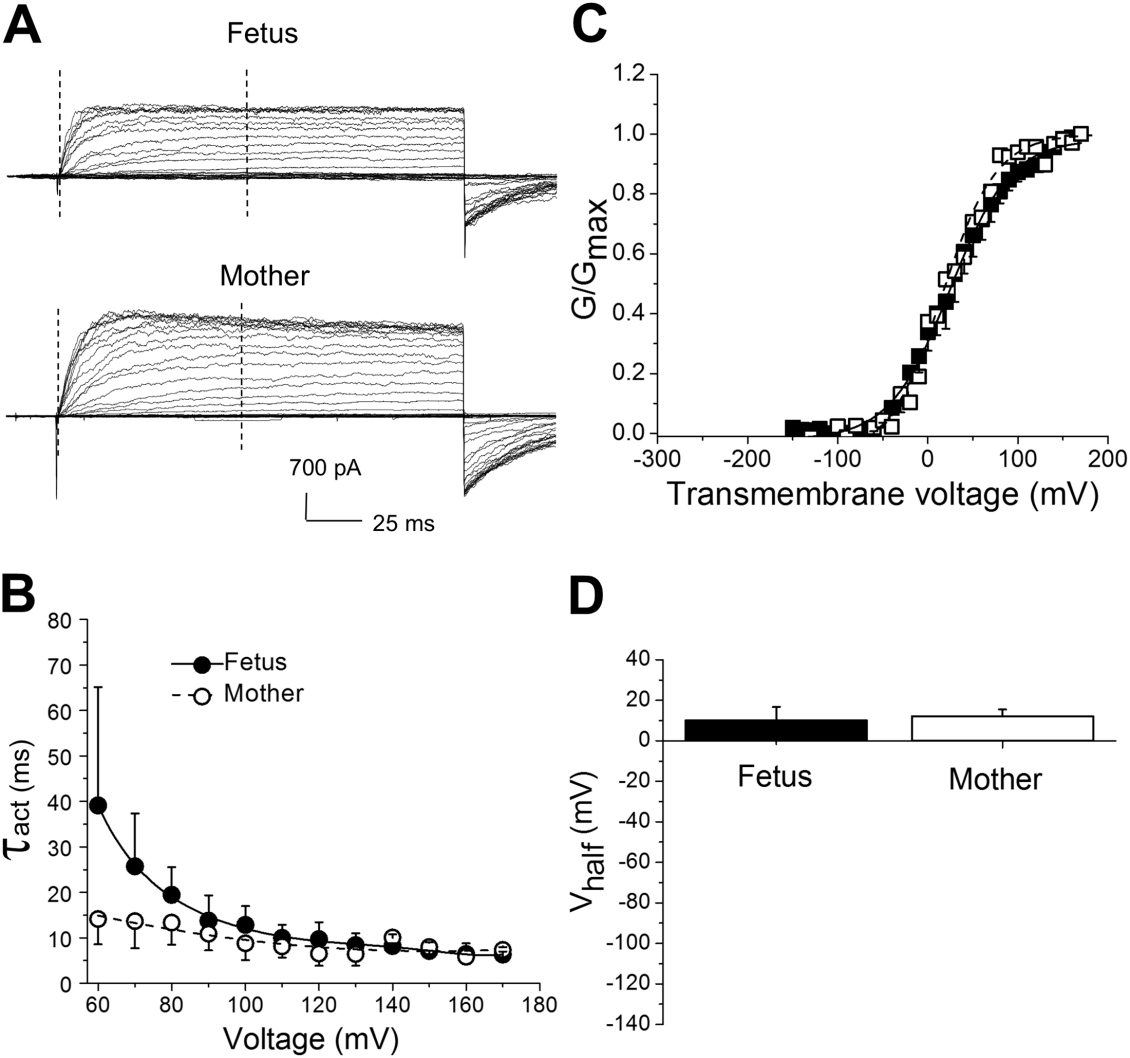
Evaluation of activation kinetics of macroscopic BK currents did not reveal significant differences between fetal and maternal membrane patches. **A.** Original records showing macroscopic BK currents obtained from excised membrane patches from fetal and maternal cerebral artery myocytes. Currents were evoked by a series of 200 ms-long voltage steps of 10 mV from -150 to 150 mV (Vholding = -80 mV). [Ca^2+^]_free_ = 3 μM. Vertical dashed lines define recording intervals at which macroscopic currents were fitted by a single-term exponential function to define τof activation. **B.** Averaged data reflecting activation kinetics (τ_act_) in fetal and maternal myocyte membrane patches. **C**. Averaged G/Gmax-V curves from maternal and fetal artery myocyte patches. Here and in **D**, each averaged data point was obtained from 5 patches from fetal cerebral artery myocytes and 4 patches from maternal cerebral artery myocytes. Each patch was obtained from a different myocyte. **D.** Averaged Vhalf (voltage that is needed to reach half-maximal current) obtained from individual fits of G/Gmax-V data from fetal and maternal artery myocytes (P = 0.9999).

Regarding channel activity and gating, the Vhalf from NPo/NPomax-V or from G/Gmax-V plots is usually taken as a global indicator of channel steady-state activity, as it results from intrinsic voltage- and calcium-gating processes. Remarkably, Vhalf values obtained from NPo/NPomax-V data at 3 μM [Ca^2+^]_free_ were similar between fetal and maternal channels: 11±9 mV and 3±5 mV, respectively (Fig 4D and 4E). Likewise, Vhalf values obtained from G/Gmax-V plots were similar between fetal and maternal channels: 10±7 mV and 12±3 mV, respectively (Fig 5A, 5C and 5D).

Examination of macroscopic current behavior also revealed that fetal and maternal currents had similar activation time constants (τ_act_) at voltages exceeding 80 mV (Fig 5A and 5B). Yet, maternal currents developed faster (smaller τ_act_) at lower transmembrane voltages (Fig 5A and 5B). Taken together, both protein detection with immunofluorescence labeling and functional patch-clamp study led us to the conclusion that BK channel protein was present in the fetal arteries, with BK current exhibiting major phenotypic features (unitary current amplitude and Vhalf) that were similar to maternal BK currents. Subtle differences in macroscopic current activation kinetics were also detected.

### Functional contribution of fetal BK channels to the regulation of cerebral artery diameter

**Fig 6:**
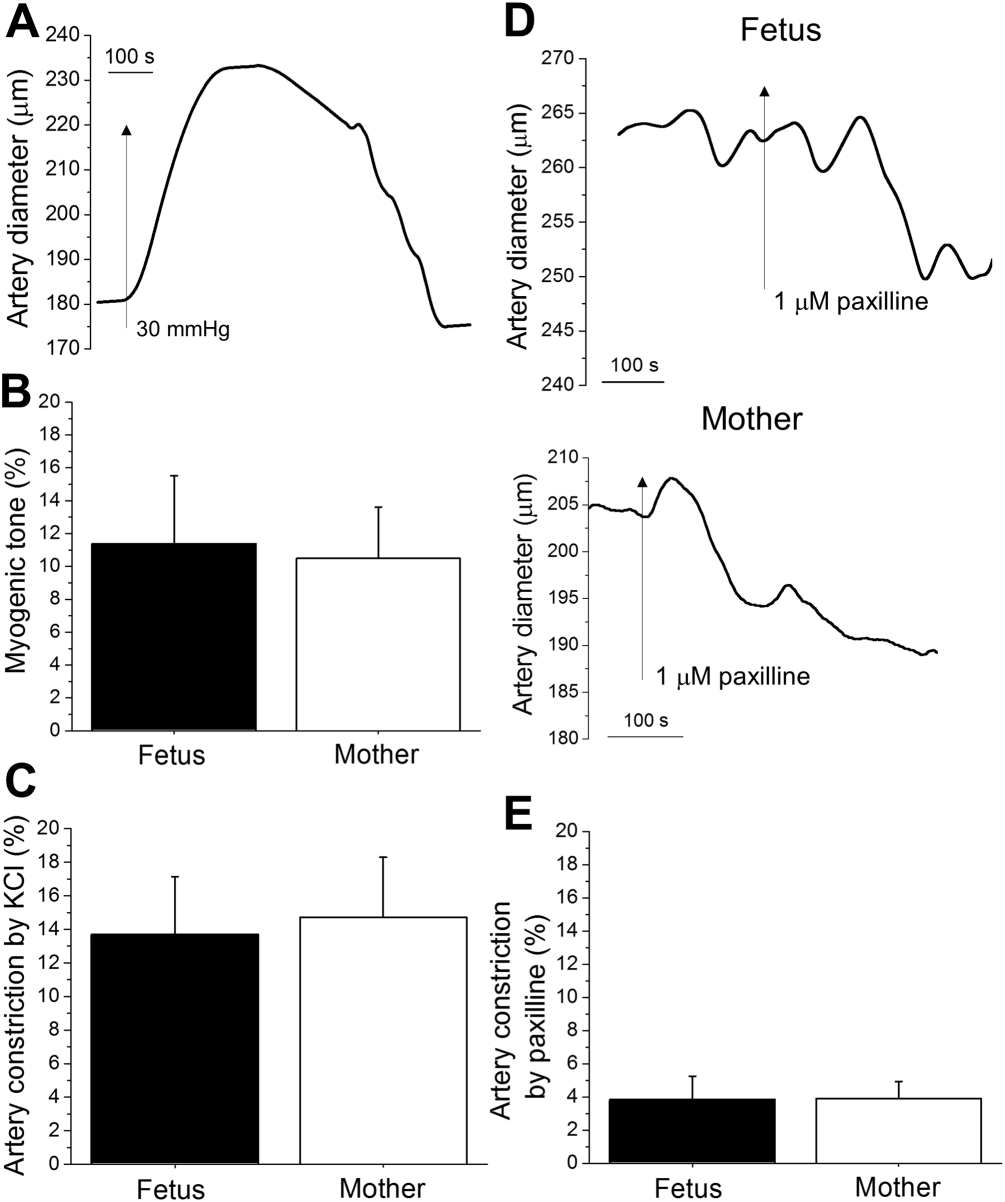
Paxilline-induced constriction of fetal and maternal cerebral arteries. **A.** Original trace of fetal cerebral artery diameter showing myogenic tone development in response to 30 mmHg of intralumenal pressure. **B.** Averaged data showing lack of difference in myogenic tone of fetal (n = 4) versus maternal (n = 4) cerebral arteries (P = 0.8676). Each artery segment was harvested from a separate animal. **C.** Averaged data showing similar degree of constriction in response to 60 mM KCl in fetal (n = 7) and maternal (n = 8) cerebral artery segments (P = 0.8393). No more than 2 artery segments were probed per individual animal donor. **D.** Original traces of cerebral artery diameter changes in response to selective BK channel blocker 1 μM paxilline following application to fetal (top record) and maternal (bottom record) *in vitro* pressurized vessels. **E.** Paxilline-induced constriction is similar in fetal (n = 6) and maternal (n = 7) arteries. Each artery recording was obtained from separate animal (P = 0.9778).

As the major role of cerebral vasculature BK channels is to control cerebral artery diameter, we used a pharmacological approach to assess fetal BK channel function at the organ level. Middle cerebral artery branches of 1^st^ and 2^nd^ order were dissected out for experimentation. Fetal and maternal middle cerebral artery branches of 270±54 μm and 246±15 μm external diameter, respectively, were pressurized *in vitro* at 30 mmHg [23]. The choice of test pressure was dictated by our earlier work, during which we observed loss of fetal cerebral artery viability when cerebral arteries of fetuses harvested during second trimester-equivalent of human pregnancy were studied at higher pressures [23]. A test pressure of 30 mmHg is on the lower side of pressure intervals that are usually faced by cerebral arteries in primates, and close to values reported for mean systolic blood pressure of baboons during pathological conditions, such as sepsis [33]. At 30 mmHg, fetal cerebral artery myogenic tone reached 11±4%, which was not significantly different from the maternal cerebral artery tone of 10±3% (Fig 6A and 6B). Constriction in response to 60 mM KCl was also similar in fetal and maternal arteries (Fig 6B). In particular, KCl-induced constriction reached on average 14±3% and 15±4% in fetal and maternal artery segments, respectively (Fig 6C).

Application of the selective BK channel blocker paxilline (1 μM) [34–35] to pressurized cerebral arteries from fetuses consistently resulted in a decrease in diameter (Fig 6D). The paxilline-induced constriction of fetal cerebral arteries was not significantly different from the constriction of maternal cerebral arteries under the same experimental conditions. This decrease reached on average 4±1% from the artery diameter before paxilline application (Fig 6E).

Interestingly, we consistently observed a faster response of maternal cerebral arteries to paxilline when compared to fetal artery segments (averaged time to the start of paxilline-induced constriction in fetal arteries was 215±75 sec versus 90±24 sec in maternal ones). This difference in the onset of paxilline effect was not statistically significant (P = 0.1143 by Mann-Whitney test). However, relatively low P prompted us to perform a power analysis: with the size of the effect and magnitude of standard deviation we reach the power of 0.8 at an alpha level of 0.05 with 23 data-points in each group. The biological basis of the potentially different timing of paxilline response between fetal and maternal arteries remains speculative (see Discussion). Regardless of interpretation, a possible differential onset of the paxilline effect is observed, and our data demonstrate a similar magnitude of paxilline-induced constriction in fetal vs. maternal cerebral arteries. We also observe a significant contribution of BK channels to the control of diameter in fetal and maternal vessels.

## Discussion

In the present work we used a baboon model of pregnancy to identify and characterize functional BK channels in fetal cerebral artery myocytes. Features indicating BK channel involvement, including large unitary conductance for K^+^, and calcium-, voltage- and paxilline-sensitive effects, were all identified. Analysis revealed that BK currents from fetal cerebral artery myocytes displayed unitary current amplitude, and Vhalf values that were similar to BK currents from maternal cerebral arteries. At the organ level, the degree of BK channel-mediated constriction of *in vitro* pressurized arteries was also similar in fetal and maternal preparations.

Investigation of developmental changes in protein expression and function not only helps in improving our knowledge of general aspects of fetal physiology, but is also driven by clinical need. In particular, the molecular pharmacology of very young ages, such as with fetal and neonatal pharmacology, has been largely understudied [36–38]. In addition to the complex and unique aspects of perinatal-neonatal pharmacokinetics [37, 39], pharmacodynamic changes associated with development may present a challenge for pharmacotherapeutic interventions. Therefore, drug targets (including ion channels) that are present and functional in adults may be absent or may have not yet reached a functional mature state, which considerably undermines their pharmacological value during the fetal and neonatal period [39–40].

Indeed, several ion channels have been reported to undergo developmental changes starting *in utero* and continuing into adulthood and senescence [17, 41–47]. Ion channel development does not always follow a linear, progressive shift towards the adult phenotype: there may be developmental “spikes” and “dives” in ionic currents along the journey to maturation [42, 45, 48]. Thus, there is no rule that can be universally applied, and the developmental time-course of ion channel maturation from one ion channel type cannot be extrapolated to another.

In the present work, we used a baboon model of pregnancy that offers major advantages over non-primate species, such as rodent and ovine models, when addressing developmental biology questions and their extrapolation to *Homo sapiens*. Baboons are evolutionarily closer to humans than rats or sheep. Moreover, baboons share critical stages of fetal development with humans [49–51]. Considering that the total length of pregnancy in baboons is 180 days—about two-thirds of the length of pregnancy in humans—the selected timing of fetal extraction via c-section at ≈165 days of gestation corresponds to near-term and perinatal period in baboons and humans, respectively [52]. Although BK channels have been previously characterized in human fetoplacental vascular and detrusor smooth muscle cells [53–54], the current work is the first report of BK channel function in cerebral artery myocytes in fetal primates, to the best of our knowledge.

We evaluated BK channel function at three different levels of integration: excised, “cell-free” patches (e.g. in the absence of freely diffusible cytosolic messengers); intact cells (in the patch-clamp cell-attached configuration); and at the organ level. Results consistently documented that the conductance under investigation had several key features of the BK (slo1) channel phenotype [5, 7, 55, 56]. In particular, channels exhibited large unitary conductance (>200 pS in symmetric K^+^ = 135 mM), which is consistent with the unitary current of BK channels when studied under a symmetric K^+^ gradient (135 mM) that was similar to ours [57]. In addition, channels were characterized by voltage- and calcium-sensitive features, and distinct pharmacology, as the current was abated by exposure to paxilline, a selective BK channel blocker [26, 34–35]. Records obtained from the cell-attached configuration, however, revealed an ionic current of smaller amplitude, this current only appearing at high transmembrane voltages (Fig 1A). This lesser amplitude may result from a subconductance of BK channels, possibly arising from differential splicing of the BK channel. This appearance of different splice variants has been described during development of the central nervous system [58]. Alternatively, the current in question might represent non-BK channel activity, such as found in a nonselective cation channel that has been characterized in rat vascular smooth muscle cells [59]. Positive identification of the current in question would require detailed permeation and gating studies, beyond the scope of the study described here.

The subunit composition of the fetal BK current in our work could not be determined with certainty. Indeed, we were unable to positively identify the presence of the BK channel beta1 and gamma protein in baboon cerebral artery myocytes due to the inability to reach satisfactory antibody performance in the baboon tissue. However, expression of *KCNMB1* gene coding BK beta1 subunit has been detected in human aorta [60]. Also, functional studies in human cerebral artery myocytes confirmed simultaneous occurrence of calcium sparks and BK currents [61], suggesting the presence of functional beta1 subunits. In the absence of BK beta1, such co-occurrence would be significantly blunted, as was demonstrated in cerebral artery myocytes of *KCNMB1* knock-out mouse [5]. Thus, it is highly likely that BK beta1 subunits are present in primate cerebral artery tissue. In addition, our PCR analysis favors the possibility of LRRC26 presence as well, although this possibility requires experimental validation at protein and functional levels.

As of now, recently reported electrophysiological data may offer some insights onto the topic of BK channel composition. It is known that BK beta1 subunits increase the apparent calcium sensitivity of the BK channel, and thereby decrease the voltage needed to reach a particular current amplitude [1, 5, 7, 11]. In the present work, we used a calcium level (3 μM) that is within the optimal interval for the functional coupling between BK alpha and beta1 subunits [56]. Vhalf values from the present data were almost identical to the values previously reported for currents generated by beta1-containing BK channels, as opposed to currents generated by homomeric channels only formed by alpha subunits [11, 56]. Electrophysiological data (Vhalf values) therefore strongly support the presence of functional beta1 subunits within the fetal cerebral artery myocyte BK channel complex. We cannot rule out that BK gamma (LRRC26) subunits are also part of the fetal cerebral artery myocyte BK channel complex. Like BK beta1, LRRC26 induces a leftward shift in G/Gmax-V and Po/Pomax-V curves to lower transmembrane voltages [6, 8]. Moreover, presence of LRRC26 protein does not preclude functional co-assembly of BK channel-forming subunits with accessory beta proteins [6, 62]. Thus, the gating shift caused by the beta subunits may be additive to one resulting from the presence of LRRC26 [62]. Moreover, a similar shift in Vhalf may be reached by channel complexes with different stoichiometry of beta and gamma subunits. This possibility becomes even more plausible considering the subtle differences detected in macroscopic current activation kinetics between fetal and maternal myocyte BK currents.

In addition to BK channel characterization by electrophysiological and biochemical means, we determined the contribution of BK current to the regulation of cerebral artery diameter using *in vitro* pressurized cerebral arteries. Under our experimental conditions, we detected paxilline-induced constriction of maternal and near-term fetal cerebral arteries. The ability of BK channel blocker to evoke measurable constriction indicates the presence of functionally active BK channels in baboon cerebral arteries. However, the response to paxilline in fetal arteries was somewhat slower to develop than was observed in maternal counterparts. The biological basis of the phenomenon remains merely speculative. Artery constriction is a multi-step event that is initiated at the level of paxilline penetration through adventitia (external) layer of the artery, involves paxilline interaction with BK channel complex, and ultimately impacts the myocyte contractile machinery. Such a multi-step event likely reveals physiological differences between fetal and maternal artery segments. Alternatively, the intriguing possibility may be suggested that this phenomenon arises from differential binding kinetics of paxilline to the fetal versus maternal BK channel. Regardless of the mechanistic basis for a differential onset of paxilline effect, the overall degree of paxilline-induced constriction did not differ between maternal and fetal arteries and reached on average a 4% reduction in diameter. This constriction is expected to result in up to 16% drop in cerebral blood flow, since flow is proportional to the artery radius to the 4^th^ power as described by Poiseuille’s equation.

Overall, although differences between fetal and maternal BK channel composition and/or function exist, our data indicate several similarities between cerebral artery BK channels of fetal and maternal origin. Ion channel expression and function in fetal versus adult cerebral arteries have been previously studied in sheep. In particular, near-term fetal cerebral arteries had greater density of L-type calcium channels when compared to adult, non-pregnant sheep [63]. With regards to BK channels themselves, the ability of the BK channel blocker iberiotoxin to constrict near-term ovine fetal cerebral arteries was reported [64], suggesting the presence of a functional BK channel in sheep. However, the calcium sensitivity of the fetal BK channel in the main branch of the fetal middle cerebral artery was higher than that of the adult non-pregnant sheep [17]. This report differs from our current data showing that baboon near-term fetal cerebral artery myocyte BK currents are characterized by Vhalf values that are similar to the maternal Vhalf values. This difference may have several causes. First, the difference may arise from species-specific developmental changes in BK channel composition and function. These developmental changes may involve not only BK channel proteins *per se* (such as differential splicing), but also their modulatory machinery. The latter includes protein kinase-driven phosphorylation of BK subunits, which results in higher calcium sensitivity of the fetal current in the ovine model [17–18]. Second, the adult group in our study was represented by pregnant baboon mothers that were approaching delivery. We cannot rule out the possibility that pregnancy might affect the BK channel activity in adult cerebral artery myocytes. Third, there is a possibility that our study suffered from a statistical type 2 error, in which false negative results obtain. Unlike many other animals used in research, baboons present a long lifespan, with diverse medical history and environmental exposures. On one hand, this fact offers the advantage that the model is likely representative of diversity in human populations. On the other hand, it introduces unusual variability in datasets that, combined with the scarce availability of baboons for research, is prone to obtaining type 2 errors in experimental results. However, achieving statistical significance with large numbers of test animals raises the question of practical significance, as the small magnitude of the detected effect may not offer practical opportunities for clinical translation.

## Conclusions

Our work, for the first time, provides the identification, characterization, and direct comparison of fetal BK channels with their maternal counterparts in cerebral artery myocytes. Based on our data at protein, ionic current, and organ (arterial diameter) levels, fetal BK channels are functional. Although subtle differences between fetal and maternal BK currents exist, fetal currents exhibit several major characteristics that are similar to those that define the adult phenotype. Thus, fetal BK channels may represent a valid pharmacological target for therapeutic interventions during the perinatal period of primate development.

## Abbreviations

BK channel: large conductance voltage- and calcium-gated potassium channel
I/O: inside out (excised patch
NPo: open probability, where N = number of channel opening levels in the patch, and Po = open probability of a single channel
Vhalf: transmembrane voltage that is needed to reach half-maximal current amplitude
